# Supplementary material for: An AI-guided screen identifies probucol as an enhancer of mitophagy through modulation of lipid droplets
Source: PLoS Biol. 2023 Mar 2;21(3):e3001977. doi: 10.1371/journal.pbio.3001977 (PMC9980794; doi:10.1371/journal.pbio.3001977)
Supplement: S1 Fig — (A) Leave-one-out cross validation and retrospective analyses were performed to evaluate the ability of the model to identify bona fide mitophagy enhancers. Similarity scores between each chemical assessed and the mitophagy enhancer training set were calculated and used to assign a rank out of 3,231 DrugBank molecules from most to least similar in ascending order. (B) Leave-one-out cross validation results were used to construct a receiver operating characteristic curve, which demonstrates the predictive performance of the model. The area under the curve for the ROC curve is 0.9513. (C) Following validation, the model was deployed to identify new mitophagy enhancers from the DrugBank candidates based on information from a wide array of sources including PubMed literature, patent filings, and biological databases. (PDF) [file pbio.3001977.s001.pdf]

A

leave one out cross validation

chemical	similarity score	rank/3231
NICOTINAMIDE	0.002995589	1
SRT1720	0.002521317	2
PIFITHRIN ALPHA	0.002929496	1
RESVERATROL	0.002306876	4
FISETIN	0.00251808	3
OLAPARIB	0.001209273	131
KINETIN	1.40327E-05	777

retrospective analysis

chemical	similarity score	rank/3231
OLAPARIB	0.001274202	126

B

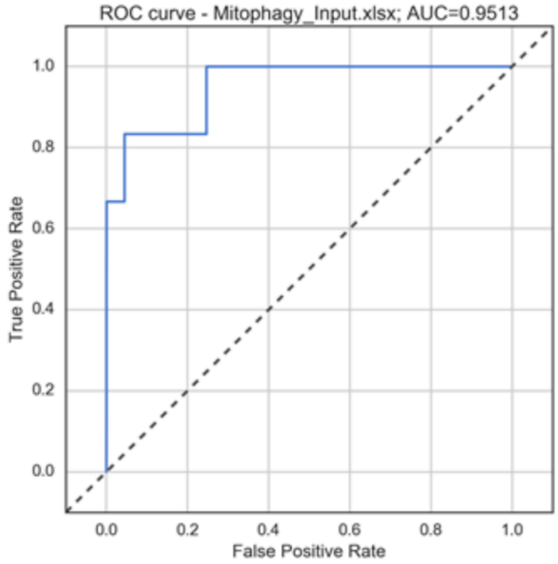

C

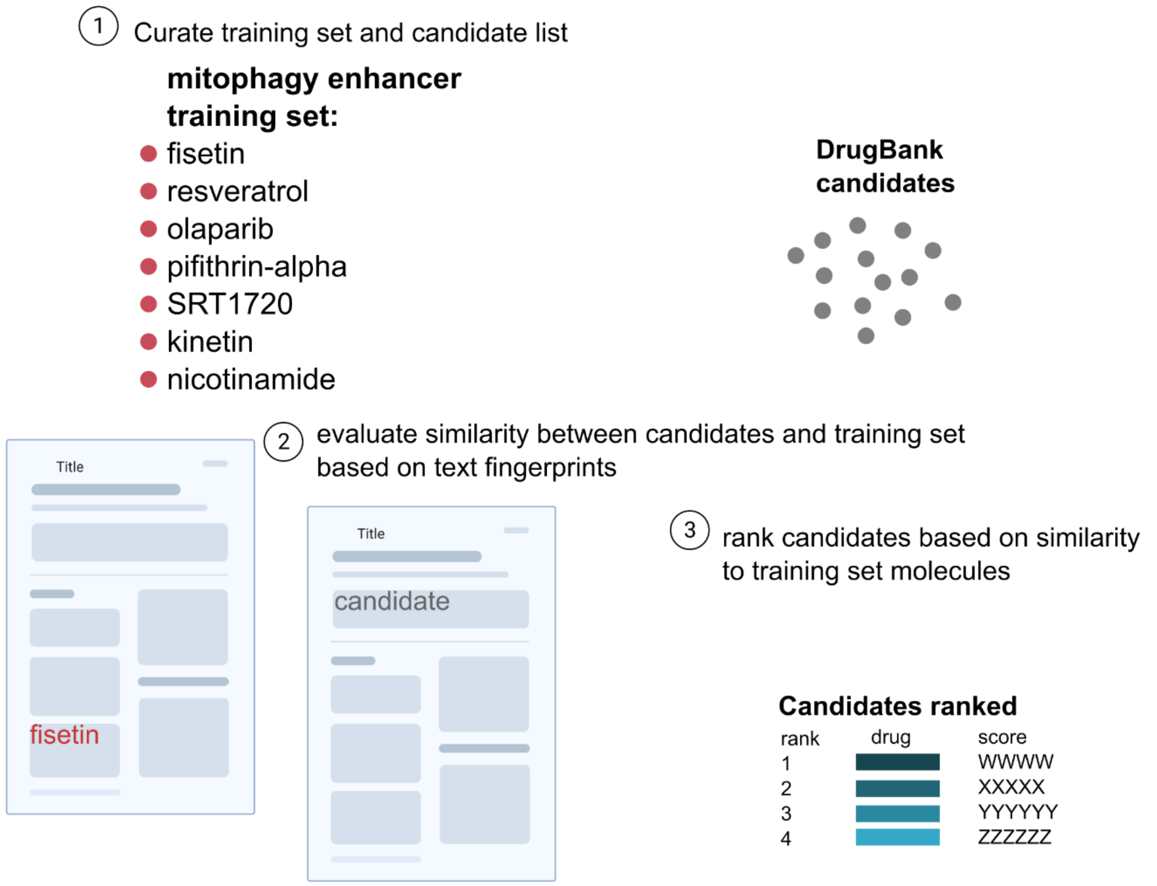

Appendix Figure S1. *in silico* screen to identify candidate mitophagy enhancers in DrugBank database of drugs amenable to repurposing

(A) Leave one out cross validation and retrospective analyses were performed to evaluate the ability of the model to identify bona fide mitophagy enhancers. Similarity scores between each chemical assessed and the mitophagy enhancer training set were calculated and used

to assign a rank out of 3231 DrugBank molecules from most to least similar in ascending order.
(B) Leave-one-out cross validation results were used to construct a receiver operating characteristic curve which demonstrates the predictive performance of the model. The area under the curve for the ROC curve is 0.9513.
(C) Following validation, the model was deployed to identify new mitophagy enhancers from the DrugBank candidates based on information from a wide array of sources including PubMed literature, patent filings and biological databases.
