# Supplementary material for: An AI-guided screen identifies probucol as an enhancer of mitophagy through modulation of lipid droplets
Source: PLoS Biol. 2023 Mar 2;21(3):e3001977. doi: 10.1371/journal.pbio.3001977 (PMC9980794; doi:10.1371/journal.pbio.3001977)
Supplement: S2 Fig — (A) Normalized MAD z-scores of 79 candidate DrugBank molecules screened in the mitochondrial clearance screen. Probucol, 3-methoxybenzamide, and lapatinib are highlighted among other compounds in descending order of rank. (B) Chemical structures of hit molecules highlighted in A. (C) HeLa cells expressing GFP-Parkin and mito-DsRed. Cells were pretreated with small molecules (1 μM) for 2 hours followed by 24-hour treatment with CCCP (10 μM) to induce prolonged mitophagy, resulting in loss of mito-DsRed signal from many cells (mitochondrial clearance). Arrows denote cells that retained mitochondrial signal. The percentage of remaining cells with no/low mito-DsRed signal was calculated as the screening readout. (D) Immunoblotting using antibodies for outer mitochondrial membrane protein VDAC1 was performed on lysates from cells pretreated with small molecule (1 μM) prior to CCCP (10 μM) time course. (E) Quantification of VDAC1 levels normalized to Ponceau staining to assess protein loading. Data information: Normalized MAD z-score values were calculated based on two independent screening replicates in A. Four independent biological replicates were performed for E. Bars represent mean values, and error bars represent SEM. * indicates p-value <0.05. Statistical analysis was performed using an unpaired two-sided Student t test to compare DMSO and probucol at each time point. The data underlying the graphs shown in the figure can be found in S1 Data and S3 Dataset. (PDF) [file pbio.3001977.s002.pdf]

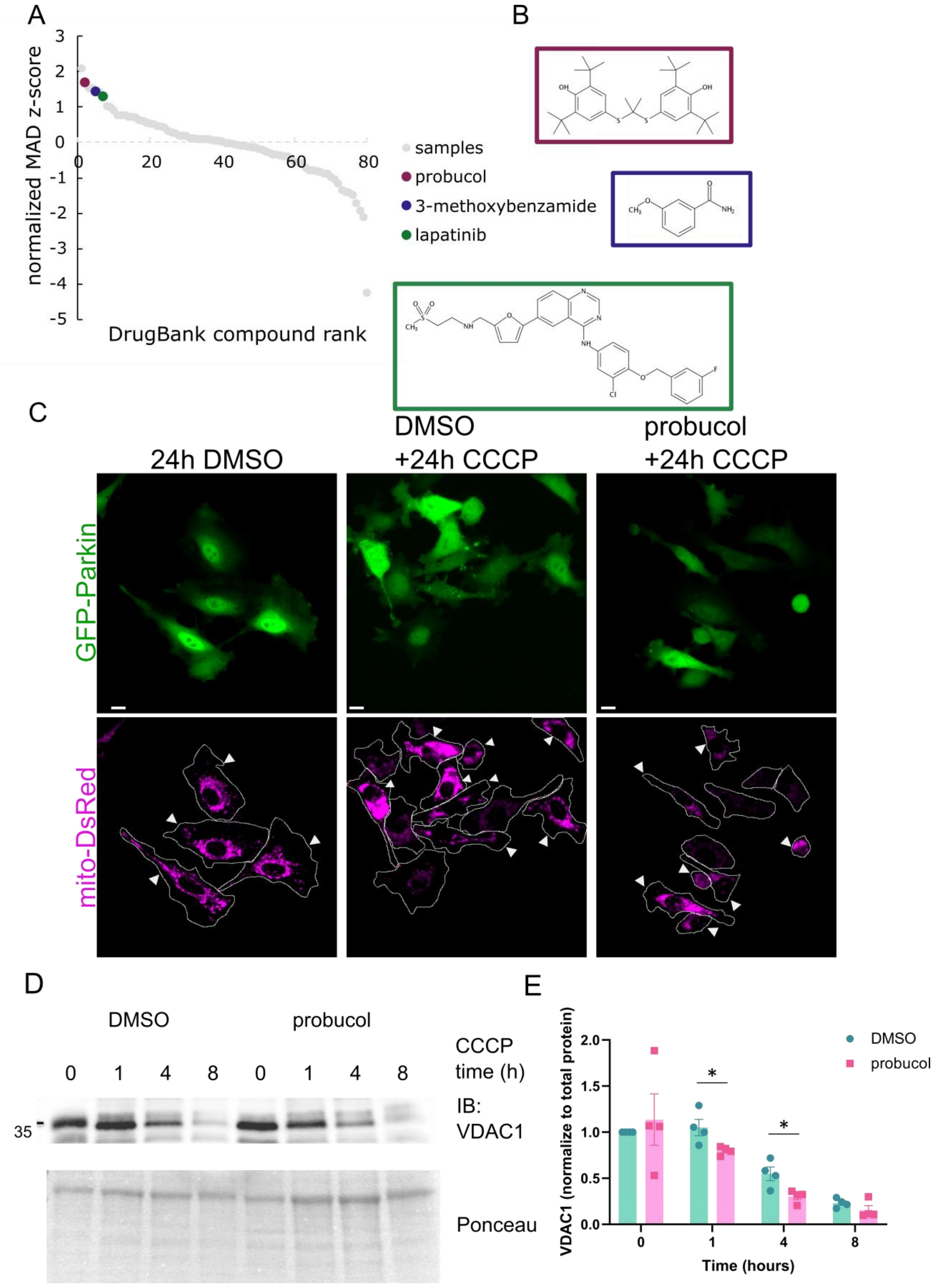
A
normalized MAD z-score
DrugBank compound rank
samples
probucol
3-methoxybenzamide
lapatinib
B
C
24h DMSO
DMSO
+24h CCCP
probucol
+24h CCCP
GFP-Parkin
mito-DsRed
D
DMSO
probucol
CCCP
time (h)
IB:
VDAC1
Ponceau
E
VDAC1 (normalize to total protein)
Time (hours)
DMSO
probucol

Appendix Figure S2. Cell-based mitochondrial clearance screen to evaluate candidates identified *in silico*

A) Normalized MAD z-scores of 79 candidate DrugBank molecules screened in the mitochondrial clearance screen. Probucol, 3-methoxybenzamide and lapatinib are highlighted amongst other compounds in descending order of rank.
B) Chemical structures of hit molecules highlighted in A).
C) HeLa cells expressing GFP-Parkin and mito-DsRed. Cells were pre-treated with small molecules (1 µM) for 2 hours followed by 24-hour treatment with CCCP (10 µM) to induce prolonged mitophagy, resulting in loss of mito-DsRed signal from many cells (mitochondrial clearance). Arrows denote cells which retained mitochondrial signal. The percentage of remaining cells with no/low mito-DsRed signal was calculated as the screening readout.
D) Immunoblotting using antibodies for outer mitochondrial membrane protein VDAC1 was performed on lysates from cells pre-treated with small molecule (1 µM) prior to CCCP (10 µM) time course.
E) Quantification of VDAC1 levels normalized to Ponceau staining to assess protein loading.
Data information: Normalized MAD z-score values were calculated based on two independent screening replicates in A). 4 independent biological replicates were performed for E). Bars represent mean values and error bars represent SEM. * indicates p-value <0.05. Statistical analysis was performed using an unpaired two-sided student’s t-test to compare DMSO and probucol at each time point.
