# Supplementary material for: An AI-guided screen identifies probucol as an enhancer of mitophagy through modulation of lipid droplets
Source: PLoS Biol. 2023 Mar 2;21(3):e3001977. doi: 10.1371/journal.pbio.3001977 (PMC9980794; doi:10.1371/journal.pbio.3001977)
Supplement: S3 Fig — (A) On Day 1, GFP-Parkin mito-DsRed HeLa cells are seeded in 96-well plates. On Day 2, cells are pretreated with 1 μM concentration of the small molecule library for 2 hours prior to the addition of 10 μM CCCP for 24 hours. Cells are then fixed and DAPI staining is performed to visualize nuclei. Cell Profiler and Cell Profiler Analyst tools are used to differentiate cells that retain mito-DsRed signal and ones with no/low mito-DsRed signal. (B) Mitochondrial clearance values for positive control wells pretreated with DMSO for 2 hours in place of small molecules and followed by 24-hour treatment with CCCP to induce mitophagy and negative control wells containing cells treated with DMSO alone. (C) Z-factor values calculated from the positive and negative control replicate wells in each of the independent biological screening replicates. The data underlying the graphs shown in the figure can be found in S1 Data. (PDF) [file pbio.3001977.s003.pdf]

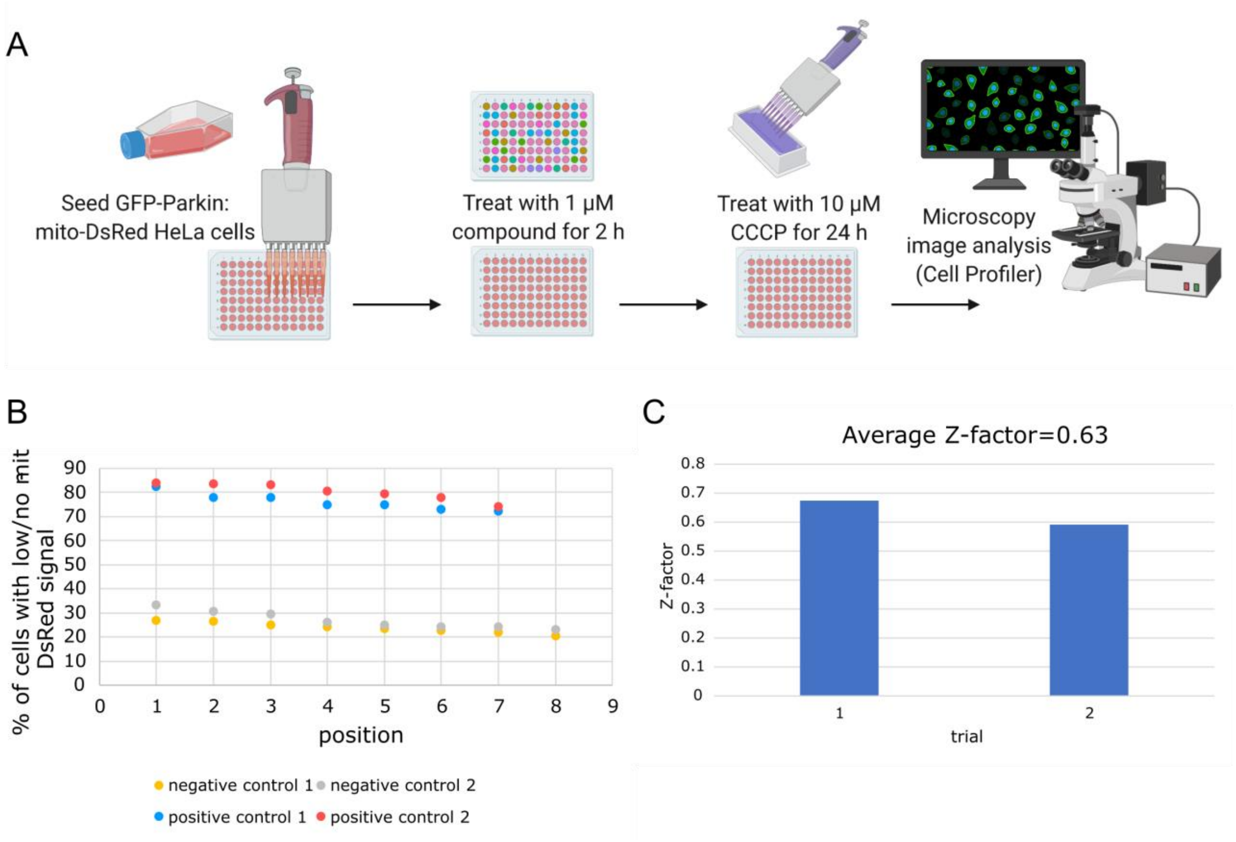

Appendix Figure S3: Mitochondrial clearance screening workflow. (**A**) On Day 1, GFP-Parkin mito-DsRed HeLa cells are seeded in 96-well plates. On Day 2 cells are pre-treated with 1 µM concentration of the small molecule library for 2 hours prior to the addition of 10 µM CCCP for 24 hours. Cells are then fixed and DAPI staining is performed to visualize nuclei. Cell Profiler and Cell Profiler Analyst tools are used to differentiate cells which retain mito-DsRed signal and ones with no/low mito-DsRed signal. (**B**) Mitochondrial clearance values for positive control wells pre-treated with DMSO for 2 hours in place of small molecules and followed by 24-hour treatment with CCCP to induce mitophagy and negative control wells containing cells treated with DMSO alone. (**C**) Z-factor values calculated from the positive and negative control replicate wells in each of the independent biological screening replicates.
