# Supplementary material for: An AI-guided screen identifies probucol as an enhancer of mitophagy through modulation of lipid droplets
Source: PLoS Biol. 2023 Mar 2;21(3):e3001977. doi: 10.1371/journal.pbio.3001977 (PMC9980794; doi:10.1371/journal.pbio.3001977)
Supplement: S4 Fig — (A) Immunoblotting whole-cell lysates using antibodies for inner mitochondrial membrane protein ATP5A. HeLa cells stably expressing GFP-Parkin were treated with the indicated drugs at 1 μM concentration in combination with 10 μM CCCP. Irrelevant lane in the center of the blot was removed for clarity, but both right and left side of blot and Ponceau correspond to the same image from the same membrane. Ponceau staining was used to visualize protein loading. (B) Densitometry analysis was performed to assess ATP5A levels normalized to Ponceau loading. The data underlying the graphs shown in the figure can be found in S1 Data. (PDF) [file pbio.3001977.s004.pdf]

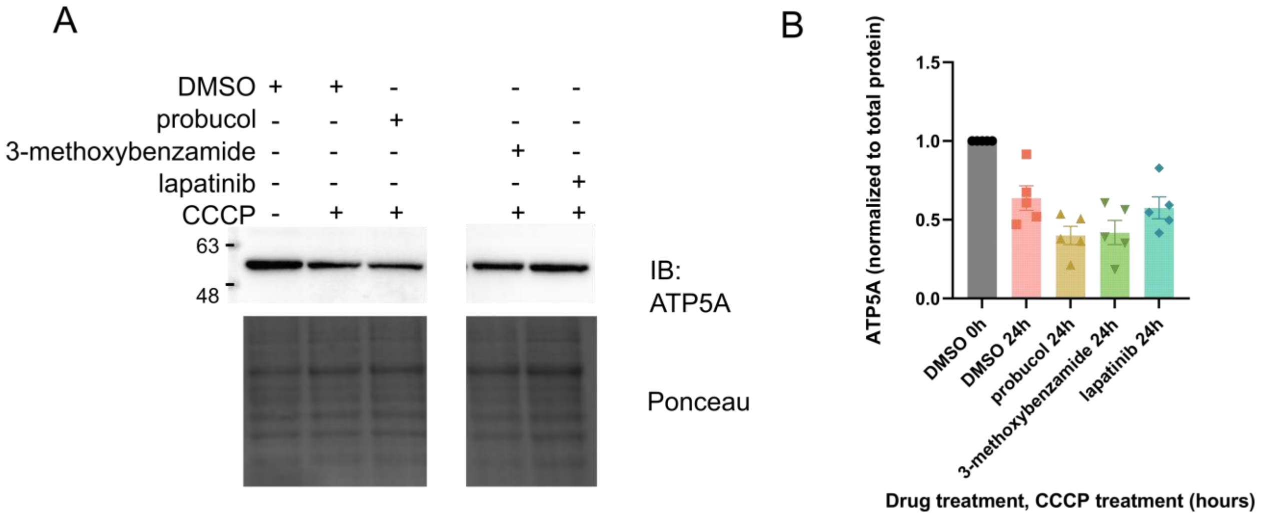

Appendix Figure S4: Immunoblotting to assess ATP5A levels following prolonged mitochondrial damage. (**A**) Immunoblotting whole cell lysates using antibodies for inner mitochondrial membrane protein ATP5A. HeLa cells stably expressing GFP-Parkin were treated with the indicated drugs at 1 µM concentration in combination with 10 µM CCCP. Irrelevant lane in the center of the blot was removed for clarity, but both right and left side of blot and Ponceau correspond to the same image from the same membrane. Ponceau staining was used to visualize protein loading. (**B**) Densitometry analysis was performed to assess ATP5A levels normalized to Ponceau loading
