# Supplementary material for: An AI-guided screen identifies probucol as an enhancer of mitophagy through modulation of lipid droplets
Source: PLoS Biol. 2023 Mar 2;21(3):e3001977. doi: 10.1371/journal.pbio.3001977 (PMC9980794; doi:10.1371/journal.pbio.3001977)
Supplement: S5 Fig — (A) HeLa cells expressing GFP-Parkin were pretreated with probucol or DMSO for 2 hours prior to 1-hour treatment with CCCP. Immunostaining with antibody specific for phospho-Ubiquitin S65 (p-Ub S65) was performed on cells. (B) The percentage of cells that are positive for mitochondrial p-Ub S65 signal and in which (C) Parkin distribution is mitochondrial was calculated for each treatment. (D) Whole-cell lysates from treatments as described in A were probed with antibody against p-Ub S65. Ponceau staining was used to assess protein loading. Three independent biological replicates were performed for all experiments and are represented by data points, and bars depict means. Error bars display SEM. ANOVA statistical analysis with Dunnett’s multiple comparison correction was performed; * and ** indicate p-value <0.05 and 0.01, respectively. The data underlying the graphs shown in the figure can be found in S1 Data. (PDF) [file pbio.3001977.s005.pdf]

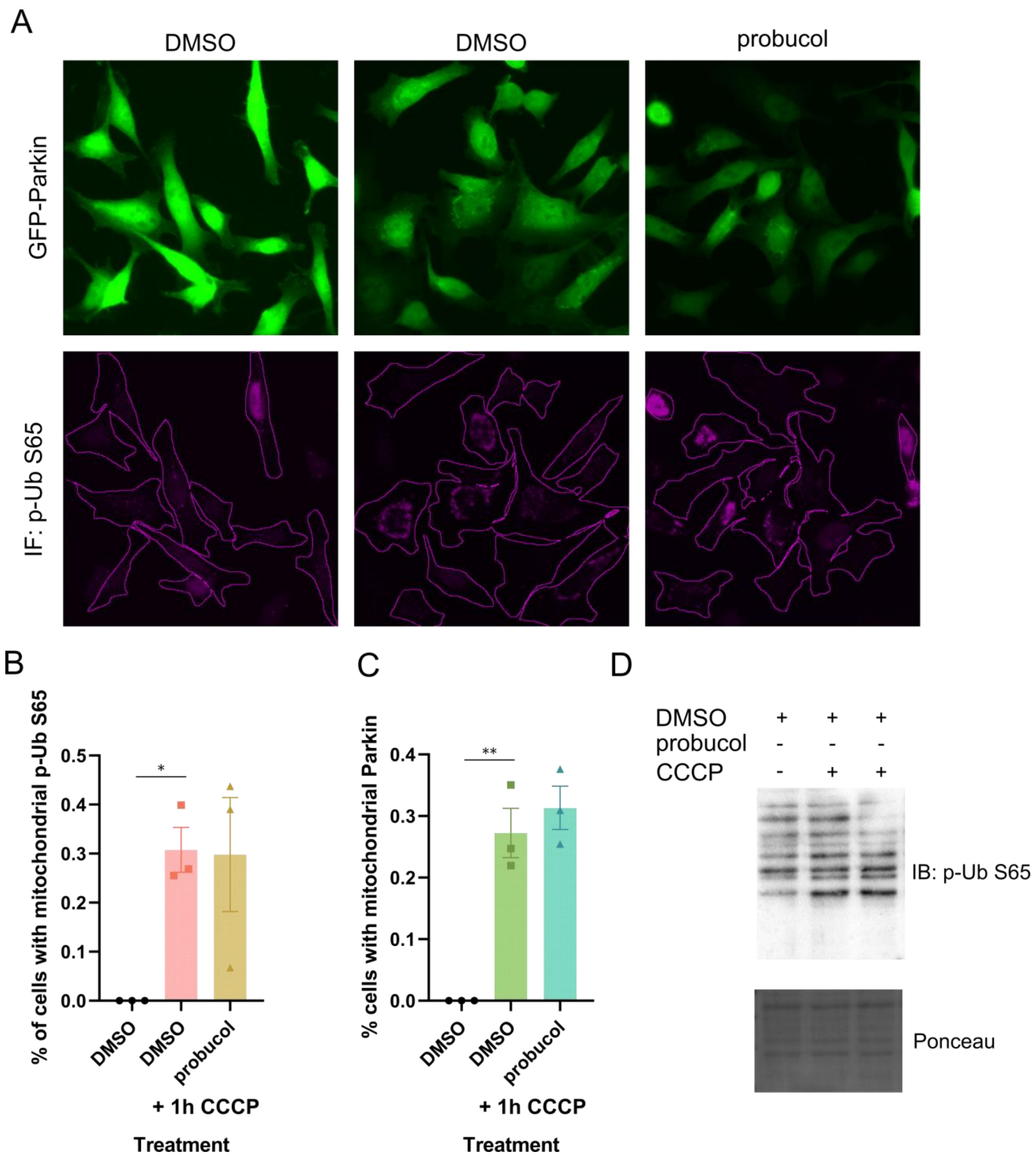

Appendix Figure S5: PINK1-mediated phosphorylation of mitochondrial ubiquitin and Parkin recruitment are not affected by probucol treatment. (**A**) HeLa cells expressing GFP-Parkin were pre-treated with probucol or DMSO for 2 hours prior to 1 hour treatment with CCCP. Immunostaining with antibody specific for phospho-Ubiquitin S65 (p-Ub S65) was performed on cells. (**B**) The percentage of cells which are positive for mitochondrial p-Ub S65 signal and in which (**C**) Parkin distribution is mitochondrial was calculated for each treatment. (**D**) Whole cell lysates from treatments as described in A) were probed with antibody against p-Ub S65. Ponceau staining was used to assess protein loading. Three independent biological replicates were performed for all experiments and are represented by data points and bars

depict means. Error bars display SEM. ANOVA statistical analysis with Dunnett’s multiple comparison correction was performed, * and ** indicate p-value<0.05 and 0.01, respectively.
