# Supplementary figures and images for: An AI-guided screen identifies probucol as an enhancer of mitophagy through modulation of lipid droplets
Source: PLoS Biol. 2023 Mar 2;21(3):e3001977. doi: 10.1371/journal.pbio.3001977 (PMC9980794; doi:10.1371/journal.pbio.3001977)

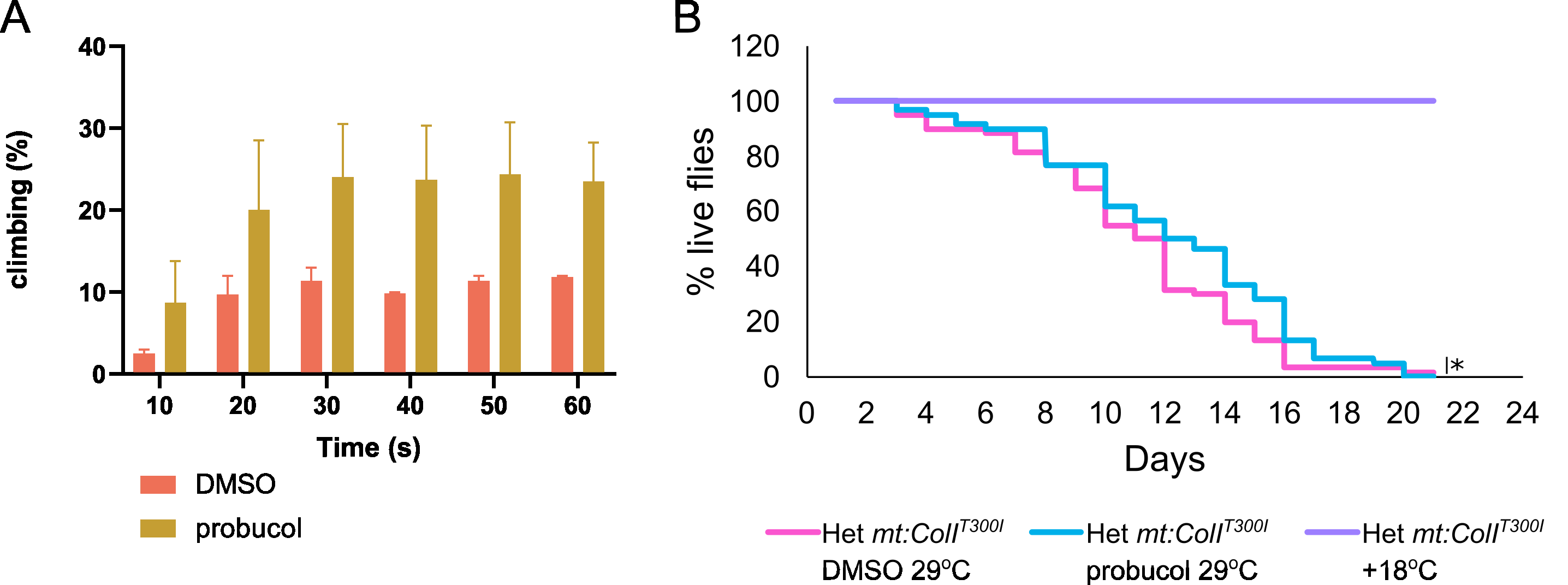

Supplement: S6 Fig — (A) The percentage of flies climbing beyond a height of 12.5 cm is displayed, in addition to (B) the survival of the flies in both groups. As a control, heteroplasmic mt:ColIT300I were maintained at permissive temperature of 18°C. Three independent biological replicates were performed for both A and B, and at least 20 flies were included in each replicate. Bars represent mean values in A, and error bars represent SEM. For A, unpaired Student t tests were performed to evaluate differences between DMSO and probucol. For B, log-rank test analysis was performed to compare the survival of the two treatment groups housed at 29°C. * indicates p-value <0.05. The data underlying the graphs shown in the figure can be found in S1 Data. (TIFF) [file pbio.3001977.s006.tiff]
