# Supplementary material for: An AI-guided screen identifies probucol as an enhancer of mitophagy through modulation of lipid droplets
Source: PLoS Biol. 2023 Mar 2;21(3):e3001977. doi: 10.1371/journal.pbio.3001977 (PMC9980794; doi:10.1371/journal.pbio.3001977)
Supplement: S7 Fig — (A) Immunoblotting was performed to assess ABCA1 levels in lysates derived from the brains of flies in which human ABCA1 transgene expression is driven by pan-neuronal elav-Gal4. (B) Immunoblotting was performed using antibodies against ABCA1 and tubulin as a loading control on HeLa cells lysates from cells transfected with either pLKO1 control or shABCA1 vectors. The blots pictured in A and B are representative of 3 independent biological replicates. (C) HeLa cells expressing the mitoQC reporter and Cerulean-Parkin were transfected with pLKO1 or shABCA1 and treated with CCCP for 6 hours. (D) Mitophagy was assessed in flies with expression of the mitoQC reporter in the presence and absence of the human ABCA1 transgene induced by the TH-Gal4 driver. Food containing the indicated combinations of probucol and paraquat was administered. (PDF) [file pbio.3001977.s007.pdf]

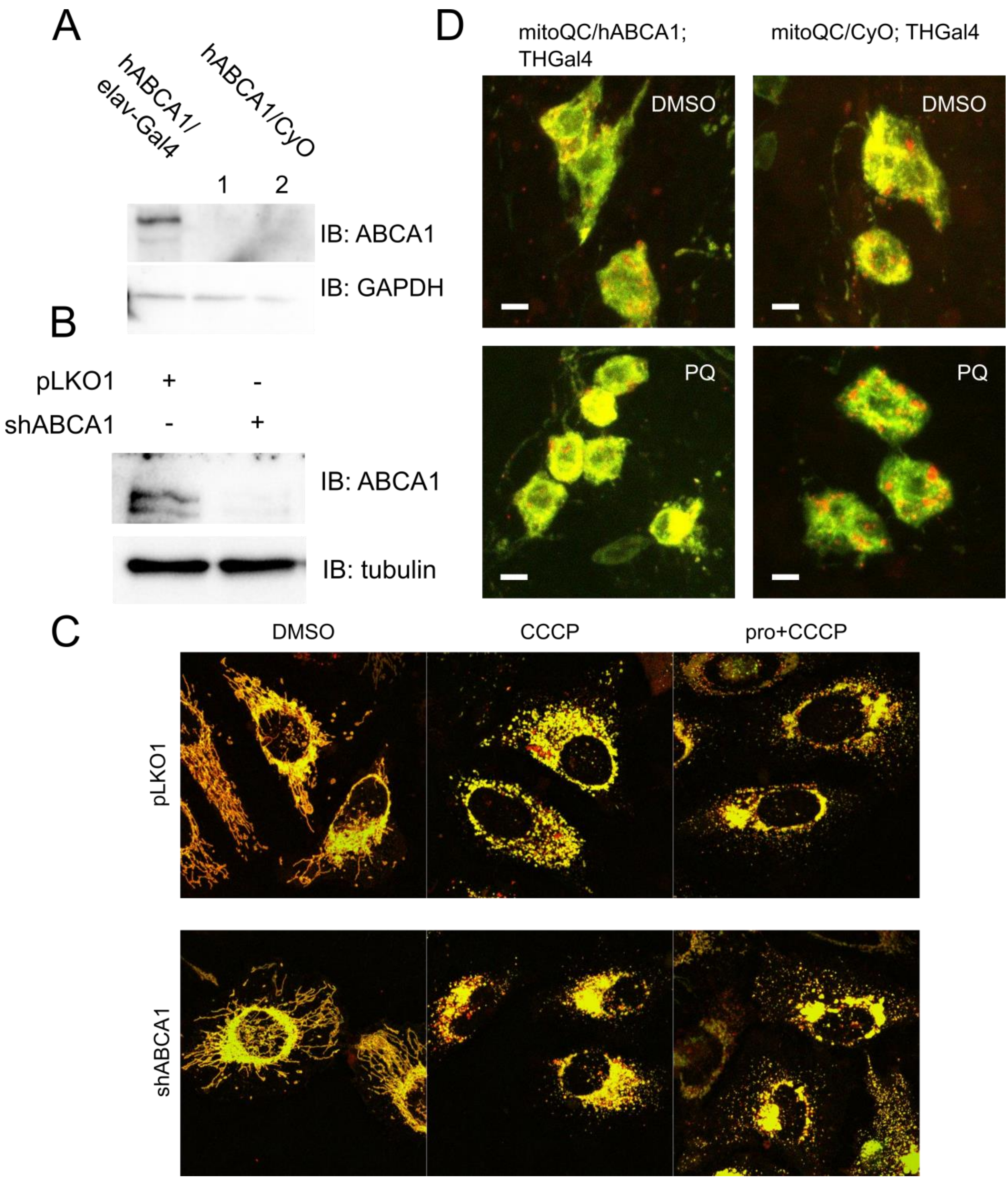

Appendix Figure S7: Effects of ABCA1 manipulations on mitophagy. (**A**) Immunoblotting was performed to assess ABCA1 levels in lysates derived from the brains of flies in which human ABCA1 transgene expression is driven by pan-neuronal elav-Gal4. (**B**) Immunoblotting was performed using antibodies against ABCA1 and tubulin as a loading control on HeLa cells lysates from cells transfected with either pLKO1 control or shABCA1 vectors. The blots pictured in A) and B) are representative of 3 independent biological replicates. (**C**) HeLa cells expressing the mitoQC reporter and Cerulean-Parkin were transfected with pLKO1 or shABCA1 and treated with CCCP for 6 hours. (**D**) Mitophagy was assessed in flies with

expression of the mitoQC reporter in the presence and absence of the human ABCA1 transgene induced by the TH-Gal4 driver. Food containing the indicated combinations of probucol and paraquat was administered.
