# Supplementary material for: An AI-guided screen identifies probucol as an enhancer of mitophagy through modulation of lipid droplets
Source: PLoS Biol. 2023 Mar 2;21(3):e3001977. doi: 10.1371/journal.pbio.3001977 (PMC9980794; doi:10.1371/journal.pbio.3001977)
Supplement: S8 Fig — HeLa cells were treated with the indicated combination of DMSO, probucol, CCCP, and DGAT inhibitors. BODIPY staining was performed to visualize LDs and immunostaining with ATP5A antibody. (PDF) [file pbio.3001977.s008.pdf]

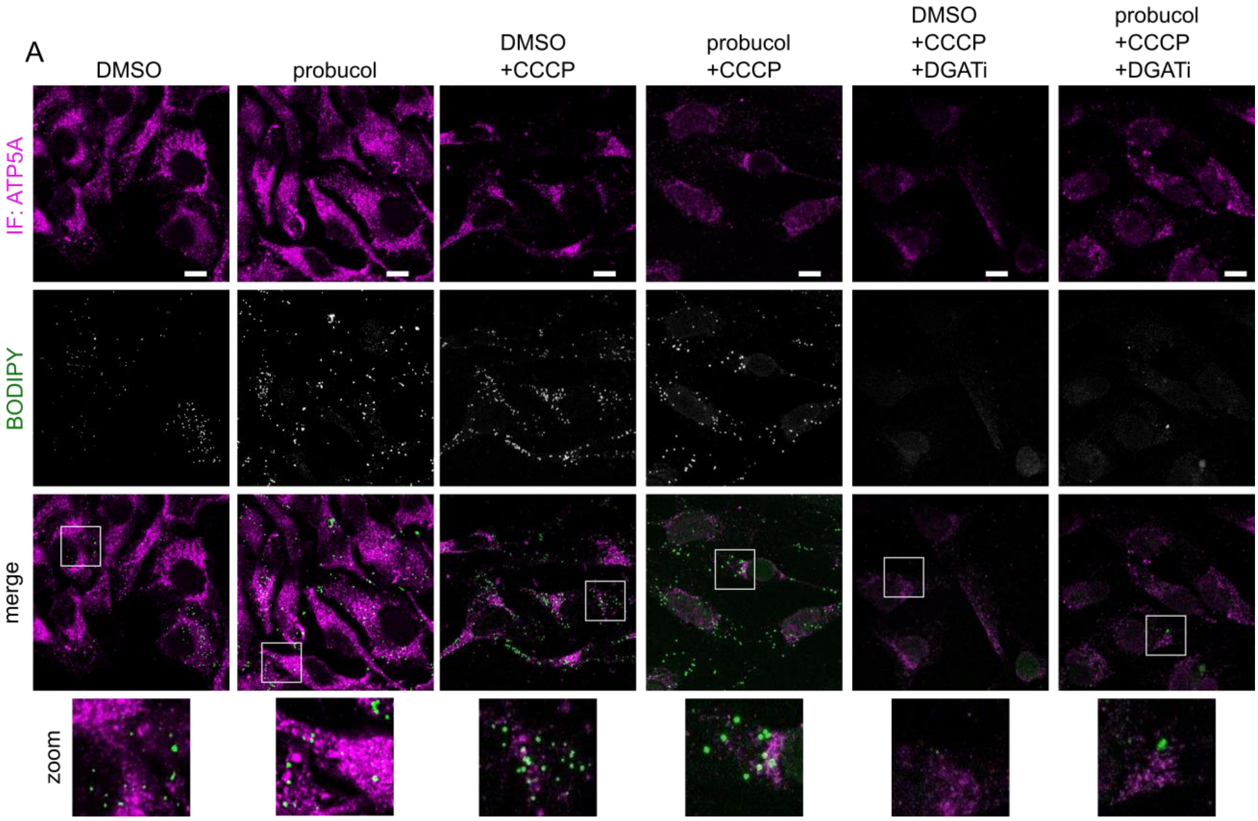

Appendix Figure S8: Lipid droplet area expansion following CCCP treatment is reduced by probucol treatment. HeLa cells were treated with the indicated combination of DMSO, probucol, CCCP and DGAT inhibitors. BODIPY staining was performed to visualize LDs and immunostaining with ATP5A antibody.
