# Supplementary material for: An AI-guided screen identifies probucol as an enhancer of mitophagy through modulation of lipid droplets
Source: PLoS Biol. 2023 Mar 2;21(3):e3001977. doi: 10.1371/journal.pbio.3001977 (PMC9980794; doi:10.1371/journal.pbio.3001977)
Supplement: S9 Fig — (A) HEK293 cells were either incubated in DMEM, DMEM with CCCP, or HBSS media for 6 hours. Lysates were separated by SDS-PAGE, and immunoblotting was performed using antibodies that recognize LC3 and GAPDH, as a loading control. (B) Densitometry was performed to measure the levels of lipidated LC3-II, which were normalized to the GAPDH loading control. Unpaired t tests were used to evaluate differences between DMSO and probucol. * indicates p-value <0.05. The data underlying the graphs shown in the figure can be found in S1 Data. (PDF) [file pbio.3001977.s009.pdf]

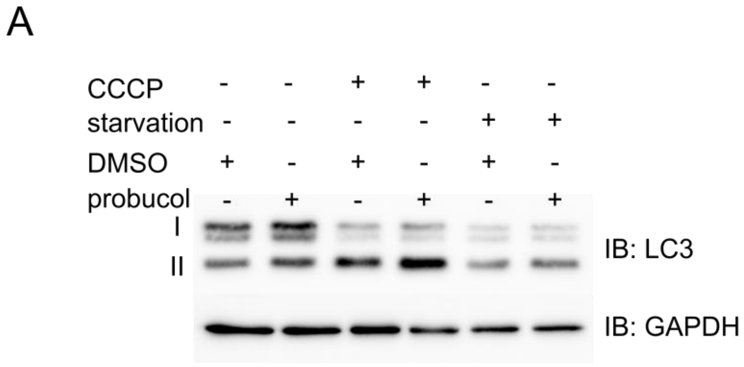

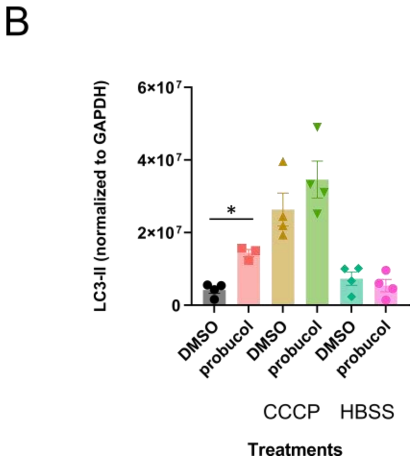

Appendix Figure S9: Effect of probucol on LC3 lipidation under basal conditions, following mitochondrial depolarization and starvation. **A**) HEK293 cells were either incubated in DMEM, DMEM with CCCP or HBSS media for 6 hours. Lysates were separated by SDS-PAGE and immunoblotting was performed using antibodies which recognize LC3 and GAPDH, as a loading control. **B**) Densitometry was performed to measure the levels of lipidated LC3-II, which were normalized to the GAPDH loading control. Unpaired t-tests were used to evaluate differences between DMSO and probucol. * indicate p-value<0.05.
